# Hypersegmentation of Granulocytes and Monocytes in a Patient with Primary Myelofibrosis Treated with Hydroxycarbamide

**DOI:** 10.4274/tjh.galenos.2019.2018.0395

**Published:** 2019-05-03

**Authors:** Monika Błocka-Gumowska, Justyna Holka, Olga Ciepiela

**Affiliations:** 1 Central Laboratory at Public Central Teaching Hospital in Warsaw, Warsaw, Poland; 2 Medical University of Warsaw, Students Scientific Group of Laboratory Medicine, Warsaw, Poland; 3 Medical University of Warsaw Department of Laboratory Diagnostics, Warsaw, Poland

**Keywords:** Granulocytes, Hypersegmentation, Hydroxycarbamide, Monocytes, Primary myelofibrosis

A 62-year-old man with a history of primary myelofibrosis was admitted to the emergency room due to abdominal pain. He remains under maintenance therapy with hydroxycarbamide. Complete blood count showed the following: white blood cell (WBC) count, 169.73x10^9^/L, including 65.8% neutrophils and 24.4% immature granulocytes; hemoglobin, 113 g/L; mean corpuscular volume, 115.20 fL; and platelet count, 119x10^9^/L. A peripheral blood film showed 10% blasts, macrocytosis, and nuclear hypersegmentation of neutrophils, basophils, and eosinophils with hypersegmented-like monocytes (Figure 1). The complete hemogram was as follows: red blood cell count, 3.02x10^12^/L; hematocrit, 34.8%; red blood cell distribution width, 14.9%; mean corpuscular hemoglobin, 37.4 pg; and mean corpuscular hemoglobin concentration, 32.5 g/dL.

Ineffective treatment with hydroxyurea (sustained hyperleukocytosis and splenomegaly) was replaced by cytarabine, 6-mercaptopurine, and interferon alpha, obtaining improvement of leukocytosis (WBC count: 21.63x10^9^/L).

Hydroxycarbamide (hydroxyurea, HU) decreases the production of deoxyribonucleotides via inhibition of ribonucleoside reductase. Cytoreductive treatment with HU often results in megaloblastic anemia and hypersegmentation of neutrophils. However, impaired segmentation of other granulocytes’ nuclei and “polymorphonuclear” monocytes remain unusual findings. While the first report of hypersegmentation of basophils and eosinophils after treatment with HU was presented by Xu [1], our finding of “hypersegmented” monocytes is the first such report worldwide.

## Figures and Tables

**Figure 1:**
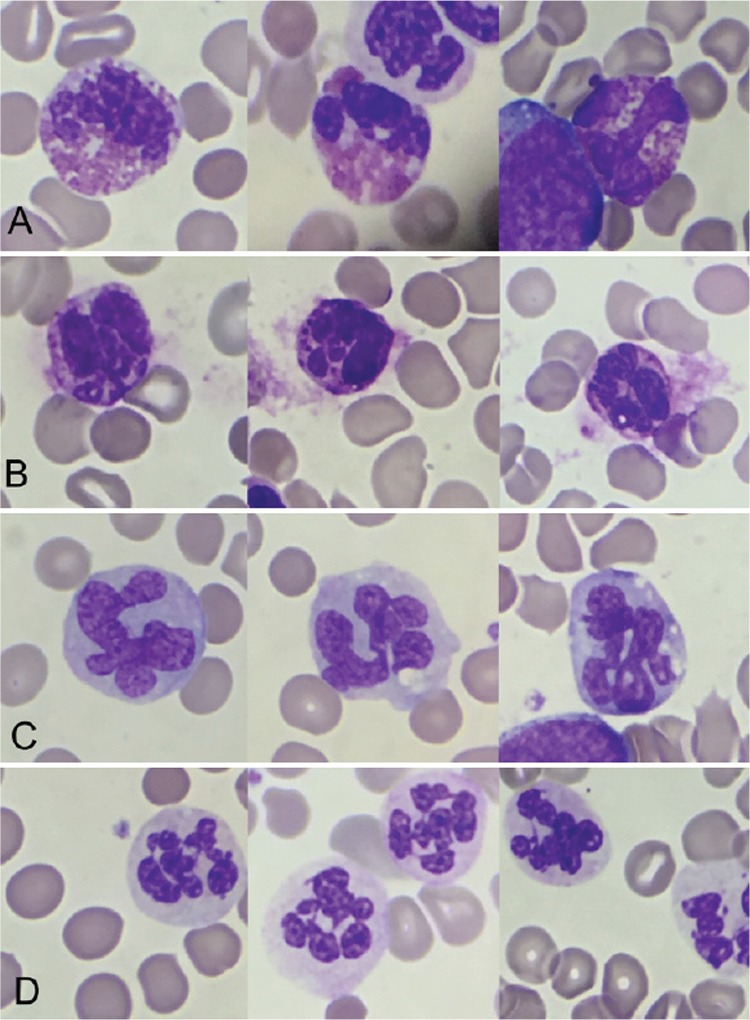
Hypersegmentation of white blood cells in peripheral blood of patient treated with hydroxycarbamide: (A) eosinophils, (B) basophils, (C) monocytes, and (D) neutrophils.
